# Speech-language Telepractice Services During the COVID-19 Lockdown: Perspectives from Parents in Malaysia

**DOI:** 10.63144/ijt.2025.6650

**Published:** 2025-06-12

**Authors:** Jing Feng, Xiao P. Choong, Pui J. Woi, Siaw C. Chai, Tian K. Quar, Jaehoon Lee, Shin Y. Chu

**Affiliations:** 1 Centre for Healthy Ageing and Wellness (H-CARE), Faculty of Health Sciences, Universiti Kebangsaan Malaysia, Kuala Lumpur, Malaysia; 2 Center for Community Health Studies (ReaCH), Faculty of Health Sciences, Universiti Kebangsaan Malaysia, Kuala Lumpur, Malaysia; 3 Centre for Rehabilitation & Special Needs Studies, Faculty of Health Sciences, Universiti Kebangsaan Malaysia, Kuala Lumpur, Malaysia; 4 Centre for Rehabilitation and Special Needs (iCaRehab), Faculty of Health Sciences, Universiti Kebangsaan Malaysia, Kuala Lumpur, Malaysia; 5 Department of Educational Psychology, Leadership, and Counselling, Texas Tech University, Lubbock, USA

**Keywords:** COVID-19, Pandemic, Parents, Speech-language services, Telepractice

## Abstract

This study explores Malaysian parents’ perspectives on speech-language pathology (SLP) telepractice services during the COVID-19 lockdown. SLP services for families living in rural areas of Malaysia can be challenging, due to travel issues associated with distance, transportation costs, scheduling conflicts, and for patients with restricted physical mobility. To investigate the efficacy of SLP services in Malaysia during the COVID-19 lockdown, 70 parents’ preferences and perspectives were explored via a survey of demographics, service engagement, and views on telepractice. During lockdown, 48.6% of children received in-person speech-language services, while 38.6% received telepractice or combined services. Of those receiving services, 91.2% reported benefits, but 70.4% disagreed that telepractice could replace in-person sessions. Parents reported children’s attention span and rapport-building challenges with telepractice. Attitudes about telepractice varied: 14.3% positive, 60% negative, 25.7% neutral. Open-ended responses revealed that some held a preference for in-person services due to attention, communication, and technological barriers. The telepractice benefits noted were parental involvement and convenience. The findings suggest a shift toward remote therapy during the COVID-19 lockdown, with positive parental perceptions. However, doubts remain about the ability of telepractice to fully replace in-person sessions, warranting further research on therapy effectiveness and limitations.

In Malaysia, limited access to speech-language pathology (SLP) services significantly impacts children in need of therapy, driven by factors such as the uneven distribution of SLP professionals and insufficient resources. Although established in the mid-1980s, SLP remains a developing profession in Malaysia, with widespread acceptance yet to be achieved (Ahmad et al., 2013). Over 850,000 children in Malaysia have disabilities; at least one-third are severe and require rehabilitation (Amar, 2008). However, the ratio of speech-language pathologists (SLPs) to the Malaysian population was calculated to be 1:100, 000 (Chu et al., 2019a). Compared to nearby countries, Malaysia lags behind. For example, Singapore has a higher SLPs-to-population ratio and better service access, while Indonesia faces similar challenges but is addressing them through community-based programs (Lee et al., 2024). This significant imbalance highlights the paucity of SLP services in Malaysia and its potential impact on patients’ access to quality care. Currently, the impact of relatively low speech-language services in Malaysia on patient care remains not widely examined. According to Tang and Chu (2023), speech-language services are primarily offered in healthcare settings and the private sector, particularly in urban regions. The geographical imbalance in the distribution of SLPs across the country is also a significant concern because it results in unequal allocation of resources and facilities throughout the country (Chu et al., 2019a; Chu et al., 2019b). Children were unable to receive speech treatment within the ‘golden window’ of speech-language development due to extensive waitlists at hospitals and clinics. This has an impact on the cost of economic development and, more importantly, welfare, wherein thousands of people access inefficient therapies.

Children with speech and language impairments require regular and frequent speech and language therapy, typically at least one weekly session with trained SLPs, which can last several weeks (Baker & McLeod, 2011; Swain et al., 2020). However, not all families receive SLP services. The reasons can be due to the shortage of available therapists leading to long waiting lists, patients’ difficulties or challenges accessing the services, and patients’ lack of understanding of the benefits of services (Chu, et al., 2019a; McLeod & Harrison, 2009; Tang & Chu, 2023). Accessing SLP services can be more challenging for families living in rural areas, due to traveling issues associated with distance, transportation costs, and scheduling conflicts, and for patients with restricted physical mobility. A potential solution to overcome these barriers and improve patients’ access to SLP services is to deliver services via telepractice (Al-Worafi, 2023). The American Speech-Language-Hearing Association ([ASHA], 2005) has defined telepractice as ‘the use of telecommunications technology to deliver professional services at a distance by linking clinician to client or clinician to clinician for assessment, intervention, and/or consultation’.

The lockdown orders during the COVID-19 pandemic compelled many healthcare providers to convert to telepractice, which not only avoided the spread of COVID-19 and ensured continuity of care, but also impacted participants’ attitudes and perceptions of this new healthcare model (Shahouzaie & Gholamiyan Arefi, 2024). According to statistics, the percentage of children receiving telepractice in New York City reached 78% by July 6, 2020, when the ban on in-home services was imposed (Kasamba et al., 2023). Similar conditions were observed in Malaysia. During the pandemic, several healthcare settings have increased telepractice services, allowing patients to access these treatments from their homes (Amaran et al., 2021; Thong et al., 2021). During the first six months of the COVID-19 pandemic, a large number of studies related to telepractice emerged, the vast majority of which reported favorable findings regarding the use of telepractice and the potential for telepractice to have a significant impact on the future of healthcare (Doraiswamy et al., 2020).

Families’ perceptions regarding the use of telepractice services have been investigated in several studies (Chen et al., 2024; Tipre et al., 2024; Wainer & Ingersoll, 2015; Wallisch et al., 2019). Those who utilized telepractice reported its accessibility and feasibility, as well as its efficacy in addressing speech and hearing issues among patients (Paul et al., 2024). From these studies, it is evident that telepractice has a beneficial impact on access to rehabilitation services for families (Brehon et al., 2023). From both the practitioners’ and researchers’ viewpoints, telepractice is a promising alternative when in-person services are limited (Ogourtsova et al., 2023). However, it is recommended to adopt a hybrid delivery approach, integrating in-person sessions in the initial stages and gradually transitioning to telepractice later (Ebrahimi et al., 2024; Paul et al., 2024). Other studies have identified several disadvantages and barriers to the use of telepractice, including difficulty in establishing rapport between clinicians and children or families, internet and technical issues, and the inability to use some materials in therapy (Ashburner et al., 2016; Fairweather et al., 2016; Paul et al., 2024; Clemens et al., 2018). These barriers could limit stakeholders’ adoption of telepractice, including patients and parents (Cole et al., 2016). Others reported that telepractice utilities should not be restricted to the COVID-19 pandemic. Telepractice should be seen as a service alternative and a potential solution for removing barriers and generating opportunities, particularly for patients and families who have difficulty accessing the service (Sahoo et al., 2023).

Although there is extensive literature on the use of telepractice to assess, diagnose, and treat various speech and communication disorders, particularly for those who have trouble obtaining these services (Keck & Doarn, 2014; Mashima & Doarn, 2008), such studies remain limited for Malaysia. One preliminary telepractice study related to treating patients with communication disorders was conducted in Malaysia, which found that using the smartphone videoconference method is feasible for delivering intensive voice therapy to individuals with Parkinson’s to gain better speech and voice functions (Chan et al., 2021). Another study was conducted at several healthcare centers that offered both audiology and speech services and found that remote services were rarely offered during the COVID-19 pandemic and most participants preferred in-person treatment. However, the majority perceived that creating awareness and training on telepractice was important (Quar et al., 2023).

The COVID-19 pandemic disrupted speech and language therapy services worldwide due to lockdowns, social distancing, and reallocation of healthcare resources. In Malaysia, the Movement Control Order (MCO: a public health measure in Malaysia to curb COVID-19, involving movement restrictions, business closures, and travel limitations) or lockdown exacerbated these challenges. Meanwhile, resource constraints and cultural or socio-economic barriers further hampered service delivery. Telepractice services were considered to play a critical role in improving access to healthcare, assisting SLPs in overcoming physical barriers to healthcare by providing access to patients and parents and minimizing discontinuity in patient care. To better understand the therapeutic practice during the pandemic, this study aimed to examine parents’ perspectives about virtual speech therapy services provided during the COVID-19 lockdown. Moreover, examining parents’ perceptions of those who have not used the service may help increase interest in and awareness of telepractice services as well as identify potential barriers that parents or families may face when using telepractice.

## Methodology

### Research Design

This study employed a cross-sectional design and was conducted at the Universiti Kebangsaan Malaysia (UKM) Clinic of Audiology and Speech Sciences (KASP). Ethical approval was obtained from the Research Ethics Committee, UKM (JEP 2022-263). Data collection occurred from June 3, 2022, to May 9, 2023, during the late phase of the COVID-19 lockdown in Malaysia. The research team distributed the survey questionnaire online via Google Forms to eligible parents attending KASP.

### Participants

A survey was distributed to 70 parents, assessing demographics, service engagement, and views on telepractice through a Google link. Participants were recruited from the University’s speech and language clinic based on the inclusion criteria: (a) parents of children diagnosed with a communication disorder, (b) parents of children who received speech-language therapy via conventional in-person services and/or online modes, and (c) parents of children aged 2–12 years. Non-Malaysian parents or those who cannot understand English were excluded. Written consent was obtained from the parents before data collection.

### Materials and Instrumentation

The survey questionnaire was developed based on a previous study (Appendix A) (Al Awaji et al., 2021). It included 19 questions, including whether the children were experiencing any speech or language impairments and whether they had received any intervention. The questionnaire also assessed parents’ perceptions of changes in service during the COVID-19 lockdown and their acceptance and willingness to participate in telepractice speech services in Malaysia. Additionally, an open-ended question solicited parental perspectives regarding the delivery of speech therapy services through telepractice.

### Data Analysis

Descriptive statistics were used to illustrate the distributions of participants’ socio-demographic characteristics and other study variables. For continuous variables, mean and standard deviation were calculated, while for categorical variables, count and frequency were reported. Chi-square test was used to compare attitudes and perceptions towards telepractice among three parent groups: Group A (those who did not receive any services during lockdown), Group B (those who only received in-person services during lockdown) and Group C (those who received telepractice or a combination of in-person services and telepractice during lockdown). The analyses were conducted using SPSS, with statistical significance set at .05 alpha level.

Open-ended responses were analyzed using the six-phase thematic analysis outlined by Braun and Clarke (2006) to ensure qualitative research integrity. The corresponding author led the analysis process, engaging in consensus coding with the second and third authors across all six stages. The authors familiarized themselves with the responses, gaining a holistic understanding of the parents’ subjective experiences. Through manual identification and coding of ideas and recurring data, related codes were organized into categories, leading to the emergence of potential themes and sub-themes. The refined and recoded data patterns were scrutinized to uncover meaningful themes. To maintain data rigor and consensus, the authors iteratively reviewed and refined the codes, categories, and themes until consensus was reached. These themes were subsequently expounded upon in the discussion section.

## Results

A total of 100 parents were recruited for this study. Following the exclusion of 30% of participants (n=30) due to personal factors, lack of interest, or feasibility issues, data from 70 parents were analyzed and described. Table 1 presents the demographic information about this sample. Most parents were mothers (77.1%, n=54), resided in the central region of Malaysia (98.6%, n=69), and held a bachelor’s degree (45.7%, n=32). The children’s ages ranged from 1 to 12 years, with the majority being aged 3–6 years (47.1%, n=33). These children experienced a variety of speech and language impairments, including difficulty in understanding requests (34.3%, n=24), limited expressive vocabulary (i.e., not able to express his/her speech) (78.6%, n=55), limited receptive vocabulary (i.e., not able to understand your speech) (35.7%, n=25) and misarticulated speech sounds (24.3%, n=17).

### Speech Therapy Experience

Table 2 presents information regarding the parents’ speech and language therapy experiences. Of the 70 parents, 94.3% (n=66) had children diagnosed with one or more speech and language disorders. Most parents (88.6 %, n=62) found a speech and language clinic in their area or city of residence, with most treatment centers being private (38.6%, n=27) and government clinics (32.9%, n=23). Most of the participating children (85.7%, n=60) received speech and language therapy, with the frequency of therapy services typically once weekly (64.3%, n=45).

### Speech and Language Therapy Services During COVID-19 Pandemic

Table 3 shows responses to questions assessing changes in speech and language services during the COVID-19 pandemic. A total of 34 (48.6%) children received speech and language therapy services during the MCO period, which spanned March 2020 to September 2021. Of these 34 children, seven received services exclusively through in person sessions, while 27 received either telepractice sessions or a combination of in-person services and telepractice sessions.

Among the parents who responded “Yes” to Q11 (Was your child receiving any speech and language therapy services during the MCO period? (n=34), 58.8% (n=20) indicated that they could maintain speech and language therapy services once a week, and 29.4% (n=10) indicated once every two weeks. A total of 31 parents (91.2%) believed their child benefited from the speech and language therapy services provided during the COVID-19 MCO period. The types of services received included in-person sessions at the speech and language clinic (20.6%, n=7), telepractice sessions only (e.g., Zoom, Google Meet, MS Teams; 55.9%, n=19), and a combination of in-person and telepractice sessions (23.5%, n=8).

**Figure 1:**
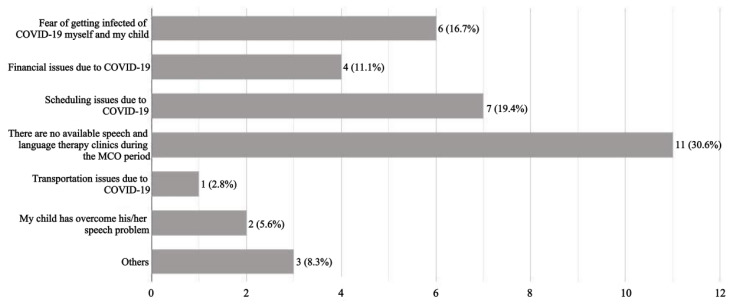
Reasons for Stopping the Speech and Language Therapy Sessions (n=36) *Note.* Others were: no improvement from the speech and language therapy sessions (n=1, 2.8%), low immunity of the child (n=1, 2.8%) or not needed for child’s age (n=1, 2.8%).

Figure 1 presents the reasons for terminating speech and language therapy sessions. Over half of the parents (51.4%, n=36) did not receive any speech and language therapy services, mainly due to unavailability of speech-language clinics during the MCO period (30.6%, n=11), scheduling issues related to COVID-19 (19.4%, n=7), and fear of getting infected with COVID-19 (16.7%, n=6).

### The Attitudes and Perceptions of Parents Towards Telepractice

Among parents of children (38.6%, n=27) who received telepractice services or a combination of in-person services and telepractice services, 70.4% (n=19) disagreed that telepractice services could replace in-person services. Similar opinions were expressed by most parents whose children did not receive any services during the MCO period (63.9%, n=23). The chi-square test found that parents’ preference for in-person services over telepractice was consistent across all groups, whether they did not receive any services (Group A), received in-person services (Group B), or received telepractice or a combination of in-person services and telepractice (Group C) during the lockdown (χ^2^(1)=0.29, p= .59). Parents expressed concerns that their children experienced a limited attention span and difficulty in building rapport during telepractice sessions. Attitudes towards telepractice varied, with 14.3% expressing positive views, 60% negative, and 25.7% neutral. The attitudes of parents (Group C) who had experienced telepractice services during the MCO period did not significantly differ from those of parents (Group A and Group B) who had not experienced telepractice services (χ^2^(2)=1.49, p= .48). Table 4 shows parents’ attitudes and perceptions of the effectiveness, applicability and usefulness of remote speech and language services.

### Qualitative Analysis

In this section, we present the findings from the qualitative analysis of parents’ opinions about having speech therapy sessions through telepractice (Q19: “What are your opinions about having speech therapy sessions through telepractice?”). A total of 37 codable open-ended responses were categorized into two themes (Table 5). In this paper, quotations from parents written in colloquial English are presented verbatim without being altered to conform to standard English conventions.

#### Theme 1: Challenges of Using Telepractice

The first theme, challenges of using telepractice, identifies four primary barriers to having speech therapy sessions through telepractice from parents’ perspectives. Twelve parents from all three groups reported that their children found it challenging to stay focused during telepractice sessions compared with in-person therapy. P16 (Group C) stated, *“kids with speech delay will have other difficulties, they will not be able to just sit down focus the screen whole time as they gave sensory difference.”* Similarly, P20 (Group C) observed that his son also showed a lack of attention during the telepractice session: *“my son having short attention as he will trying to turn into the YouTube”.* P45 (Group A) shared a similar concern: *“… when she (daughter) sees her face on the screen, she always plays with her face and can’t give attention in class.”*

The second challenge that emerged during telepractice sessions was the communication gap between children and therapists, which parents perceived could have negatively impacted the effectiveness of speech therapy. The primary impediments to effective communication stem from non-verbal cues, such as body language, gestures, or eye contact, as well as the lack of immediate, in-person services feedback. For instance, P13 (Group C) stated, *“…it (telepractice) could be somewhat difficult, since body language also plays a role in communication, and visibility is limited through telepractice...”.* Similarly, P27 (Group C) mentioned, *“it is more difficult for the therapist to build rapport with the children for engagement”*. P56 (Group A) said, *“eye contact and interaction cannot be achieved through telepractice.”*

Furthermore, three parents identified home environmental disturbances as a significant challenge in conducting speech therapy sessions at home. They emphasized that various environmental factors, including the presence of other household members, particularly children, can disrupt the therapeutic process and negatively impact its effectiveness. Inadvertent interruptions during sessions can lead to breaks in therapy flow, thereby reducing overall efficacy. P27 (Group C) stated, *“…it can be a distraction when the child is in a comfort or more familiar place”*. P34 (Group B) mentioned, *“Difficult if having more than one child in the house”.* The similar comment was stated by P3 (Group C): *“tak bagus jika ada anak kecil ia mengganggu sesi terapi”* (*“it is not good if there are other young kids disturbing the therapy session”*).

In addition, two parents reported technological constraints as the primary difficulty in utilizing telepractice services. Poor internet connections and inadequate devices are key factors that negatively impact the quality and consistency of telepractice interactions, thereby posing significant challenges. P31 (Group B) reported, *“…technical issues such as poor connection and delay in audio reception disrupt the child’s attention and at times irritate the child.”* P11 (Group C) stated, *“…lack of facilities at home.”*

#### Theme 2: Benefits of Telepractice

The second theme, “benefits of telepractice” highlights several advantages identified by parents regarding the delivery of speech therapy sessions remotely. None of the parents in Group B provided positive feedback regarding the benefits of having speech therapy sessions through telepractice. One key benefit highlighted by some parents from Groups A and C is their optimized involvement, as they need to work harder to support their children during sessions, leading to greater engagement and understanding of the therapeutic process. For example, P69 (Group A) responded, *“this telepractice therapy may help many parents speed up their child’s ability to speak.”*

Some parents have indicated that telepractice offers benefits in terms of time and logistics. By eliminating the need for travel to therapy sessions, telepractice offers considerable convenience to families. For instance, P18 (Group C) reported, *“during MCO period, it’s much more practical and effective that the session held by telepractice due to movement restrictions.”* P67 (Group A) appreciated the *“convenience and saving time for travelling (when using telepractice)”.*

Parents used telepractice as a temporary solution during the pandemic, acknowledging its usefulness in such circumstances. In total, seven parents reported embracing telepractice as a viable option during lockdown. P21 (Group C) stated, *“all time we can’t attend in-person services therapy so have to choose telepractice.”* P9 (Group C) said, *“it’s only fine during this pandemic time…”* Despite accepting telepractice as a temporary solution, some parents still consider in-person sessions the best choice. P12 (Group C) highlighted, *“while the telepractice medium is beneficial during the pandemic so as to keep the therapy momentum on-going and consistency to the child receiving the therapy, there is no doubt that in-person session would still be most effective for results.”*

Furthermore, three parents from Group C suggested a hybrid model that combined in-person services and telepractice sessions, along with enhanced parental coaching, to maximize the effectiveness of speech therapy services. For example, a parent suggested (P27), *“…it would be helpful if parents were provided with weekly guidelines, worksheets, or tasks for telepractice to work efficiently.”* According to P11, *“telepractice should be implemented via hybrid (in-person services and telepractice).”* P18 suggested, *“during the endemic phase, the session should be done in-person services or hybrid, which is in-person services and telepractice considering the limited time and far location for the session.”*

## Discussion

This study explored parents’ perspectives about speech and language services provided via telepractice during the COVID-19 lockdown period. Most of the parents were mothers. In Malaysia, women predominantly shoulder the responsibilities of childcare and household upkeep, which explains their more pronounced involvement in speech-language service participation compared to fathers and siblings (Joginder Singh et al., 2011).

The age of 3–6 years is widely recognized as a critical period for children’s language development (US Department of Health & Human Services, 2017) and an optimal timeframe for early intervention (Black et al., 2015; Brumbaugh & Smit, 2013). In our study, most children fell within this age range, indicating a considerable awareness among parents regarding the significance of early detection and intervention during this critical language development phase. However, it is noteworthy that 28.6% of the children received speech-language intervention after the age of six years for unclear reasons. This occurrence may be attributed to variations in parental awareness of language disorders, disparities in professional knowledge, or economic circumstances. This observation underscores the need to enhance public awareness regarding typical language developmental milestones and associated symptoms, alongside providing increased support information (Chu et al., 2019b). Thus, parents can be better prepared to seek suitable support and access essential services during the early stages for the attainment of optimal results.

### Speech Therapy Experience

In this study, the majority of parents reported the availability of speech and language therapy services within their residential areas or cities, potentially linked to the predominance of parents residing in the central regions of Malaysia where the surveys were distributed. This accessibility aligns with the concentration of speech-language services in the capital city of Kuala Lumpur, and the predominant practice of SLPs in major urban areas (Chu et al., 2019a). Consequently, compared to remote and underserved regions, urban areas offer more abundant and comprehensive speech-language therapy resources.

Children diagnosed with speech and language impairments frequently exhibit concomitant attention deficits (Ebert & Kohnert, 2011; El Sady et al., 2013; Manley & Wilder, 2024), a condition that poses a significant challenge to speech rehabilitation. Research suggests that the optimal strategy to address this problem may be continuous, frequent and individualised interventions (Ebert & Kohnert, 2011; Law et al., 2004). However, the current study found that most children received speech-language therapy services at a weekly frequency, which falls short of the literature’s recommended two-three sessions per week for children with speech sound disorders (Baker & McLeod, 2011). The reasons for this lower treatment frequency are unclear and may be linked to time and economic constraints. It is therefore foreseeable that children’s speech-language skills may not reach their full potential as a result of insufficient treatment frequency, which could lead to a vicious cycle of parents misperceiving their child’s treatment as ineffective and, in turn, potentially discontinuing speech-language therapy services.

This study’s distribution of private centers and government clinics was relatively balanced. Private centers and government clinics have both advantages and drawbacks. The high cost of private speech therapy interventions and assessments often hinders parents from ensuring consistent treatment of their children (Chu et al., 2019a). Due to financial constraints and limited healthcare resources, parents rely on government speech-language services through medical insurance programs and often experience prolonged waiting times (Chu et al., 2020), further delaying their children’s diagnosis and intervention.

### Speech and Language Therapy Services During COVID-19 Pandemic

In this study, nearly half of the children received speech-language therapy services during the COVID-19 pandemic, with most opting for telepractice sessions, or a hybrid of in-person and telepractice sessions. However, over half of the children did not receive speech-language therapy during the MCO period due to the limited availability of speech-language services in clinics and hospitals. This highlights the nascent state of telepractice services in Malaysia, which are yet to fully integrate and gain widespread adoption within the speech-language assessment and treatment framework. Moreover, some families remain unfamiliar with or hesitant about this emerging treatment modality.

The COVID-19 pandemic accelerated the development of telepractice services, especially in developed countries, where nations like the United States, Australia, Canada, and the European Union increasingly adopted it for language rehabilitation since January 2020 (Macoir et al., 2021). They have provided national legal, policy, and technological support for relevant practices (Theodoros, 2011). In Malaysia, telepractice requires professional training and government guidelines for effective regulation (Bani Hani et al., 2023). In circumstances where in-person interactions are not feasible, telepractice provides convenience and additional patient options. Many parents hesitate to use telepractice due to a lack of support and knowledge, leading to doubts about its effectiveness and influencing their attitudes (May & Erickson, 2014).

### The Attitudes and Perceptions of Parents Towards Telepractice

In this study, parents displayed varying attitudes and perceptions towards telepractice services. One key theme that emerged from the analysis was that 14.3% of parents, based on their open-ended responses, regarded telepractice as a viable and effective approach for treating speech-language disorders in children. They recognized its time-saving, safe, convenient, and efficient advantages, especially for families with a pressing need for speech-language therapy to mitigate the negative impact of the pandemic (Harkey et al., 2020; Singh et al., 2022; Wainer & Ingersoll, 2015; Wallisch et al., 2019). Parents in Groups A and C highlighted the benefit of increased involvement through remote sessions, which improved their understanding of the therapy and potentially sped up their children’s speech development. Such observations align with prior research underscoring the critical role of parental involvement in enhancing the outcomes of speech and language interventions (Sugden et al., 2016).

Another quarter of the parents were neutral about telepractice, acknowledging both its strengths and limitations. They emphasized the importance of tailoring therapy to their children’s needs, favoring a hybrid model that combines in-person and telepractice sessions. A hybrid model offers the benefits of enhanced patient experience and therapeutic engagement through in-person sessions, while telepractice ensures consistency in training and reduces social interactions, creating a balanced and effective approach (Quinn et al., 2021). Parents also highlighted several challenges associated with telepractice, including home distractions, such as familiar surroundings and the presence of other children. These challenges underscore the importance of establishing quiet, dedicated spaces during telepractice sessions. Additionally, the success of remote therapy hinges on reliable high-speed internet, which is a critical infrastructure requirement. To address these issues and enhance engagement, strategies such as clear verbal communication, visual aids, and interactive participation are essential.

More than half of the participating parents expressed a negative stance towards telepractice services. They disagreed with the proposition that telepractice sessions could adequately replace in-person services and opposed using telephone consultations as a substitute. These results are consistent with previous studies (Lam et al., 2021; Macoir et al., 2021), reporting that most parents found telepractice services to fall short of the standards set by traditional in-person therapy. Telephone consultations were perceived as even less acceptable than telepractice sessions (Wentink et al., 2018), as they lack non-verbal cues making it harder for SLPs to build trust and effectively conduct therapy with children (Caffery et al., 2024; Pettinari & Jessopp, 2001).

This study identified several reasons why telepractice services are often viewed negatively by parents. Children with speech-language disorders often exhibit shared attention deficits, further complicating the implementation of telepractice (Baker et al., 2023; Watson et al., 2013). Consistent with prior studies that highlight the critical role of nonverbal communication in speech therapy (Baker et al., 2023; Bayati & Ayatollahi, 2023), parents in this study were also concerned about the limited visibility of nonverbal cues and difficulties in building therapeutic relationships that could hinder effective communication between therapists and children. In contrast, in-person therapy offers significant advantages, including higher-quality personalized interactions, immediate feedback, and stronger emotional support-factors essential for the effective treatment of speech-language disorders (Ashburner et al., 2016; Fairweather et al., 2016; Marziali et al., 2006; White & Dorman, 2001; Yoo et al., 2021). This preference for traditional in-person communication remains the primary reason telepractice services have not gained widespread acceptance among parents, who continue to favor in-person sessions for the assessment and treatment of speech-language disorders.

The differing attitudes towards telepractice services can be attributed to factors such as regional culture, telepractice infrastructure, participant perspectives, and the characteristics of the service recipients (Aljabri et al., 2023; Chen et al., 2024; Seebacher et al., 2024; Tang et al., 2024). A nationwide survey conducted by the Iranian Speech Therapy Association involving 600 SLPs reported a generally positive attitude towards telepractice (Mansuri et al., 2022). Similarly, in Croatia, over 70% of SLPs with telepractice experience indicated high levels of acceptance (Kraljević et al., 2020). However, in Hong Kong, parents predominantly preferred on-site speech and language therapy over telepractice (Lam et al., 2021). In India, most parents were highly satisfied with telepractice services, their preferences were mixed when comparing telepractice to in-person sessions for both themselves and their children (Arunachalam, 2022). These varied attitudes reflect the complex interplay of cultural expectations, technological support, and individual preferences, underscoring the importance of considering regional and demographic contexts when implementing telepractice services.

## Conclusions

This study highlights the limitations of the speech-language services in Malaysia, which affect parents’ perceptions and attitudes towards telepractice services. While most children with speech disorders can receive early speech-language interventions, the average treatment frequency is once a week, which falls short of the recommended treatment frequency in the literature. During the COVID-19 lockdown, as in-person services were unavailable, telepractice services were integrated into the speech-language therapy. However, it is important to note that telepractice services did not achieve 100% coverage. Overall, parents’ experiences and acceptance of telepractice services were inferior to in-person treatment, with a slightly higher acceptability than telephone consultations.

Parents’ attitudes towards telepractice services are mixed, with nearly half acknowledging the advantages of reduced time and financial costs associated with travel and the potential for better sharing of medical resources. Many preferred a hybrid approach combining telepractice and in-person sessions, suggesting that telepractice services in Malaysia should complement rather than fully replace traditional methods. However, qualitative analysis revealed several factors contributing to reduced satisfaction with telepractice services. These include children’s limited attention spans during remote sessions, difficulties in establishing rapport between therapists and children, and technical issues such as poor internet connectivity. Parents also expressed concerns about the lack of nonverbal communication cues, which are crucial for effective speech-language therapy. Addressing these challenges by improving technical support, enhancing practitioner training, and providing tailored treatment plans may help improve parents’ perceptions of telepractice services and enhance the overall quality of speech-language therapy in Malaysia.

## Clinical Implications

Previous research and the findings of this study indicate that telepractice services in Malaysia can potentially be integrated into speech-language therapy systems as a complementary approach to in-person services. The focal point of telepractice services may be parental guidance rather than direct intervention with the child. Through telepractice, professionals can empower parents to provide speech-language parent-child interaction interventions and establish a family centered service model (Ashburner et al., 2016; Joginder Singh et al., 2011; Kocyigit et al., 2024). Telepractice services should not be limited to the interactions between SLPs and patients. They can also facilitate collaboration between SLPs and other professionals, enabling SLPs to engage in multidisciplinary or interdisciplinary team management (Joginder Singh et al., 2016). Whether parents’ acceptance of and attitudes toward telepractice services would improve or change when offered in these forms is a question that requires further research after the end of the MCO period.

## Future Recommendations

It is evident that telepractice services in Malaysia are still facing pressing issues. First, the implementation of enhanced technical support and more engaging virtual therapy methods is crucial to optimize overall effectiveness. Second, addressing issues related to maintaining focus during online therapy sessions is essential, particularly for children with attention difficulties. Furthermore, certain technical concerns raised by parents, such as alterations in speech sound characteristics resulting from remote transmission, can affect patients who require voice therapy (Weerathunge et al., 2021). Therefore, technological solutions must be developed to address this challenge.

## Limitations

Findings from a single study may not be generalizable to other populations or contexts as all participants in this study identified as Malaysian. Factors including socioeconomic status, cultural differences, health care systems’ challenges, and access to SLP services can be more challenging for families living in rural areas associated with the distance and transportation costs. Scheduling conflicts may influence the experiences and perceptions of the parents in this particular study. Thus, limiting the applicability of the findings. Furthermore, participants could have provided socially acceptable responses, which could affect the validity and reliability of the collected data. Also, there is volunteer bias associated with participants who volunteer for research studies versus those do not, leading to potential bias (American Psychological Association, 2018b; Marinos et al., 2022).

## Figures and Tables

**Table 1:** Demographics of Parents (n=70)

Characteristics	n	%
**Parents’ relationship to the child**		
Father	16	22.9
Mother	54	77.1
**State of Residence**		
Central Region of Malaysia (KL, Kajang, Selangor)	69	98.6
Eastern Region of Malaysia (Pahang)	1	1.4
**Highest level of education**		
Doctoral Degree	4	5.7
Master’s Degree	7	10
Bachelor’s Degree	32	45.7
Diploma	16	22.9
STPM	1	1.4
SPM	9	12.9
SRP	1	1.4
**Child’s age (y/o)**		
1–3	17	24.3
3;06–6	33	47.1
6;10–9	14	20
10–12	6	8.6
**Speech and Language Impairments of the Children**		
Difficulty understanding requests	24	34.3
Limited receptive vocabulary	55	78.6
Limited expressive vocabulary	25	35.7
Misarticulated speech sounds	17	24.3
Cleft lip/palate speech	3	4.3
Feeding/swallowing issue	2	2.9
Stuttering	2	2.9
Voice problem	2	2.9
Speech delay	2	2.9
Hearing loss	3	4.3
Apraxia of Speech & Autism	2	2.9
Others	3	4.3

*Note 1*. In Malaysia, STPM denotes pre-university level certificate (Sijil Tinggi Persekolahan Malaysia, completed 6.5 years of high school education), SPM stands for Malaysian certificate of high school education level (Sijil Pelajaran Malaysia, completed 5 years of high school education), and SRP represents Malaysian secondary school education level (Sijil Rendah Pelajaran, completed 3 years of high school education). *Note 2*. Others were: limited two-way communication (n=1, 1.4%), still at the pre-verbal stage (n=1, 1.4%), or difficulty in narrating a story (n=1, 1.4%).

**Table 2:** Speech and Language Therapy Experience (n=70)

Characteristics	n	%
**Does your child have any speech and language impairments at present?**		
No	4	5.7
Yes	66	94.3
**Is there a speech and language clinic available in your area/city?**		
No	8	11.4
Yes	62	88.6
**Is your child currently receiving any speech and language therapy service?**		
No	10	14.3
Yes	60	85.7
**Where are you currently receiving speech and language therapy service?**		
Private Center	27	38.6
Private Hospital	1	1.4
Government Clinic	23	32.9
Government Hospital	10	14.3
Private Home-Based	2	2.9
Attend to both Government Clinic and Hospital	1	1.4
Attend to both Government and Private Clinic	2	2.9
No Response	4	5.7
**How frequently does your child receive speech and language therapy sessions currently?**		
Not currently receiving speech therapy	4	5.7
First visit	1	1.4
Once a week	45	64.3
Once every 2 weeks	8	11.4
Once every 3 weeks	2	2.9
Once a month	3	4.3
Once every 2 months	2	2.9
Once every 3 months	2	2.9
Once every 6 months	2	2.9
Twice per student semester	1	1.4

**Table 3:** Speech and Language Therapy Services during COVID-19 Pandemic

Characteristics	n	%
**Was your child receiving any speech and language therapy service during the MCO period (March 2020-September 2021)? (n=70)**		
No	36	51.4
Yes	34	48.6
**If yes, how frequent was your child’s speech and language therapy session? (n=34)**		
Once a month	1	2.9
Once a week	20	58.8
Once every 2 weeks	10	29.4
No response	3	8.8
**Do you feel that your child benefited from the service provided by the speech therapist during the COVID-19 MCO period? (n=34)**		
No	3	8.8
Yes	31	91.2
**What kinds of speech and language services delivery were your child receiving during COVID-19 MCO period? (n=34)**		
In-person sessions at the speech and language clinic only	7	20.6
In-person sessions and consultation through telephone	2	5.9
In-person sessions and telepractice sessions	3	8.8
In-person sessions, telepractice sessions, and consultation through telephone	2	5.9
Telepractice sessions and consultation through telephone	1	2.9
Telepractice sessions only (i.e., Zoom, Google Meet, MS Teams)	19	55.9

**Table 4:** Parents’ Attitudes and Perceptions Towards Telepractice

Characteristics	GROUP A (n=36)		GROUP B (n=7)		GROUP C (n=27)		Total (n=70)	

	n	%	n	%	n	%	n	%
**In your opinion, the speech and language in-person services can be replaced by telepractice sessions. (7 no respond)**								
Agree	8	22.2	0	0	4	14.8	12	19
Do not agree	13	36.1	0	0	14	51.9	27	42.9
Totally agree	5	13.9	0	0	4	14.8	9	14.3
Totally disagree	10	27.8	0	0	5	18.5	15	23.8
**In your opinion, the speech and language in-person services can be replaced by consultation through telephone. (7 no respond)**								
Agree	2	5.6	0	0	3	11.1	5	7.9
Do not agree	18	50	0	0	15	55.6	33	52.4
Totally agree	0	0	0	0	1	3.7	1	1.6
Totally disagree	16	44.4	0	0	8	29.6	24	38.1
**What are your opinions about having speech therapy sessions through telepractice?**								
Positive opinions	6	16.7	0	0	4	14.8	10	14.3
Negative opinions	23	63.9	5	71.4	14	51.9	42	60
Neutral opinions	7	19.4	2	28.6	9	33.3	18	25.7

*Note.* GROUP A: did not receive any services during lockdown. GROUP B: only received in-person services during lockdown. GROUP C: received telepractice or a combination of in-person services and telepractice during lockdown.

**Table 5:** Themes and Subthemes from the Open-ended Responses

Theme	Subthemes
**Challenges of using telepractice**	“*It is difficult for my kids to focus:*” lack of attention
	Communication barriers
	Home environmental disturbance
	Technology limitation
**Benefits of telepractice**	“*Parents need to work harder to support:”* optimized parent’s involvement in telepractice
	Time and logistic saving
	“*It’s a good option…*”: temporary acceptance due to pandemic restrictions
	Suggestions of a combination of hybrid mode and parental coaching

## Appendix A Survey

### Section A - General information

1. Parents’s relationship to the child (Q1)
  - Father /Mother
2. City of residence (Q2)
  - KL/other________
3. Your highest level of education (Q3)
  - Primary education/SRP/SPM/STPM/Diploma/Degree/Masters/Ph.D.
4. Child’s age (Q4) Please fill in________

### Section B: Your child’s condition and speech therapy experience

1. Does your child have any speech and language problems at present? (Q5)
  - Yes/No
2. Please choose your child’s speech and language problems (Q6): (please tick whatever applicable)
  - Difficulty understanding requests
  - Has limited receptive vocabulary (i.e., not able to understand your speech)
  - Has limited expressive vocabulary (i.e., not able to express his/her speech)
  - Misarticulates speech sounds
  - Stuttering
  - Has cleft lip/palate issue
  - Has a voice problem
  - Has hearing loss
  - Has feeding/swallowing issue
  - Others. Please specify _____________
3. Is there a speech and language clinic available in your area/city? (Q7)
  - Yes/No
4. Does your child currently receive any speech and language therapy service? (Q8)
  - Yes/No
5. Where are you currently receiving speech and language therapy service? (Q9)
  Please indicate the name of the center:____________
6. How frequently does your child receive speech and language therapy sessions currently? (Q10)
  - Once a week
  - Once every 2 weeks
  - Once every 3 weeks
  - Others. Please specify___________
7. Was your child receiving any speech and language therapy service during the MCO period (March 2020-September 2021)? (Q11)
  - Yes (Go to question 12, 13, 14, 15)/No (Go to question 16)
8. If yes, how frequently was your child’s speech and language therapy sessions? (Q12)
  - Once a week
  - Once every 2 weeks
  - Once every 3 weeks
  - Others. Please specify___________
9. When was your child’s last speech and language therapy session? (Q13)
  - Please specify (Month/year):_____________
10. Do you feel that your child benefits from the service provided by the speech therapist during the COVID-19 MCO period? (Q14)
  - Yes/No
11. What kinds of speech and language service delivery was your child receiving during COVID-19 MCO period? (Q15)
  - Face-to-face sessions at the speech and language clinic only (if choose this, skip question 17 and 18).
  - Telehealth sessions only (i.e., zoom, googleMeet, MSTEAM)
  - Consultation by speech therapist through telephone only
  - Face-to-face sessions and telehealth sessions
  - Face-to-face sessions and consultation through telephone
  - Telehealth sessions and consultation through telephone
  - Face-to-face sessions, telehealth sessions, and consultation through telephone
12. Why were the speech and language therapy sessions stopped? (Q16) (Please tick 1 or more applicable choice)
  - Fear of getting infected of COVID-19 myself and my child
  - Low immunity of my child
  - Transportation issues due to COVID-19
  - Financial issues due to COVID-19
  - Scheduling issues due to COVID-19
  - There are no available speech and language therapy clinics during the MCO period
  - My child has overcome his/her speech problem
  - My child did not show any improvement from the speech and language therapy sessions
  - My family was under quarantine order
  - Others. Please specify___________
13. In your opinion, speech and language face-to-face services can be replaced by telehealth sessions (Q17)
  - Totally agree
  - Agree
  - Do not agree
  - Totally disagree
14. In your opinion, the speech and language services provided face-to-face can be replaced by consultation through telephone (Q18)
  - Totally agree
  - Agree
  - Do not agree
  - Totally disagree

### Section C: Open-ended question

1. What are your opinions about having speech therapy sessions through Telehealth? (Q19).
  ________________________
